# Left triangular ligament lesions are likely hepatic in origin

**DOI:** 10.1259/bjr.20230231

**Published:** 2023-10-03

**Authors:** Siddharth Agarwal, Rahul Munyal, Aloysious Aravinthan, Christopher Clarke

**Affiliations:** 1 Department of Radiology, Nottingham University Hospitals NHS Trust, Nottingham, United Kingdom; 2 School of Biomedical Engineering and Imaging Sciences, King’s College London, London, United Kingdom; 3 NIHR Nottingham Biomedical Research Centre, Nottingham University Hospitals NHS Trust and University of Nottingham, Nottingham, United Kingdom; 4 Nottingham Digestive Diseases Centre, Translational Medical Sciences, School of Medicine, University of Nottingham, Nottingham, United Kingdom

## Abstract

**Objectives:**

The left lobe of the liver is connected to the left hemi-diaphragm by the left triangular ligament (LTL) which crosses the left upper quadrant (LUQ) of the abdomen. LTL lesions are rare and can cause diagnostic confusion. We encountered a hepatocellular carcinoma in the LTL of one patient and given that the LTL has been shown to contain liver tissue in cadaveric studies, we hypothesise that LTL lesions are likely hepatic in origin. The aim of this case series and systematic review was to develop a list of pathological processes encountered at the LTL, which could impact differential diagnoses of lesions within the LUQ.

**Methods:**

A case series of two patients are presented. MEDLINE, EMBASE, PubMed, and Google Scholar were searched for studies published until September 2022. All case reports of LTL lesions were eligible for inclusion. PROSPERO registration: CRD42022368250.

**Results:**

Twelve out of 166 screened studies were included, with 14 cases in total. Seven described mass lesions of hepatic origin, of which three were initially misdiagnosed as gastric tumours leading to considerable diagnostic delay and additional procedures. Seven other cases described postoperative bile leaks from aberrant biliary ducts in the LTL.

**Conclusions:**

All mass lesions observed in the LTL were hepatic in origin, and all iatrogenic injuries described resulted in bile leaks.

**Advances in knowledge:**

To reduce the burden of diagnostic delay and unnecessary procedures, we would suggest that these observations support the addition of hepatic lesions to the differential diagnosis of LUQ lesions.

## Introduction

**Figure 1.**
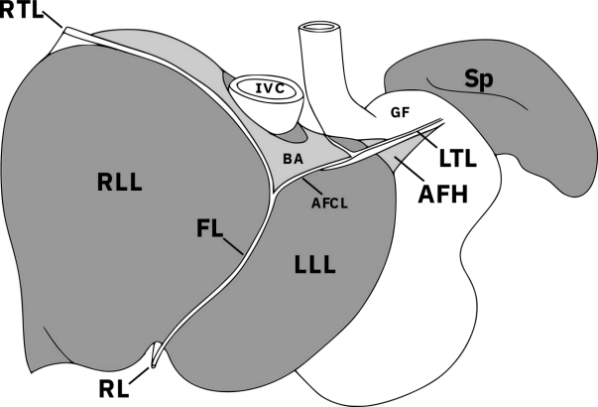
Ligaments of the liver and their relation to the other upper abdominal viscera adjacent to the diaphragm. The left triangular ligament and appendix fibrosa hepatis are continuous with the left hemi-diaphragm and are therefore often adjacent to the gastric fundus. *LTL* left triangular ligament, *AFH* appendix fibrosa hepatis, *AFCL* anterior fold of the coronary ligament, *FL* falciform ligament, *RL* round ligament, *RTL* right triangular ligament, *RLL* right lobe of liver, *LLL* left lobe of liver, *BA* bare area, *IVC* inferior vena cava, *GF* gastric fundus, *Sp* spleen.

**Figure 2.**
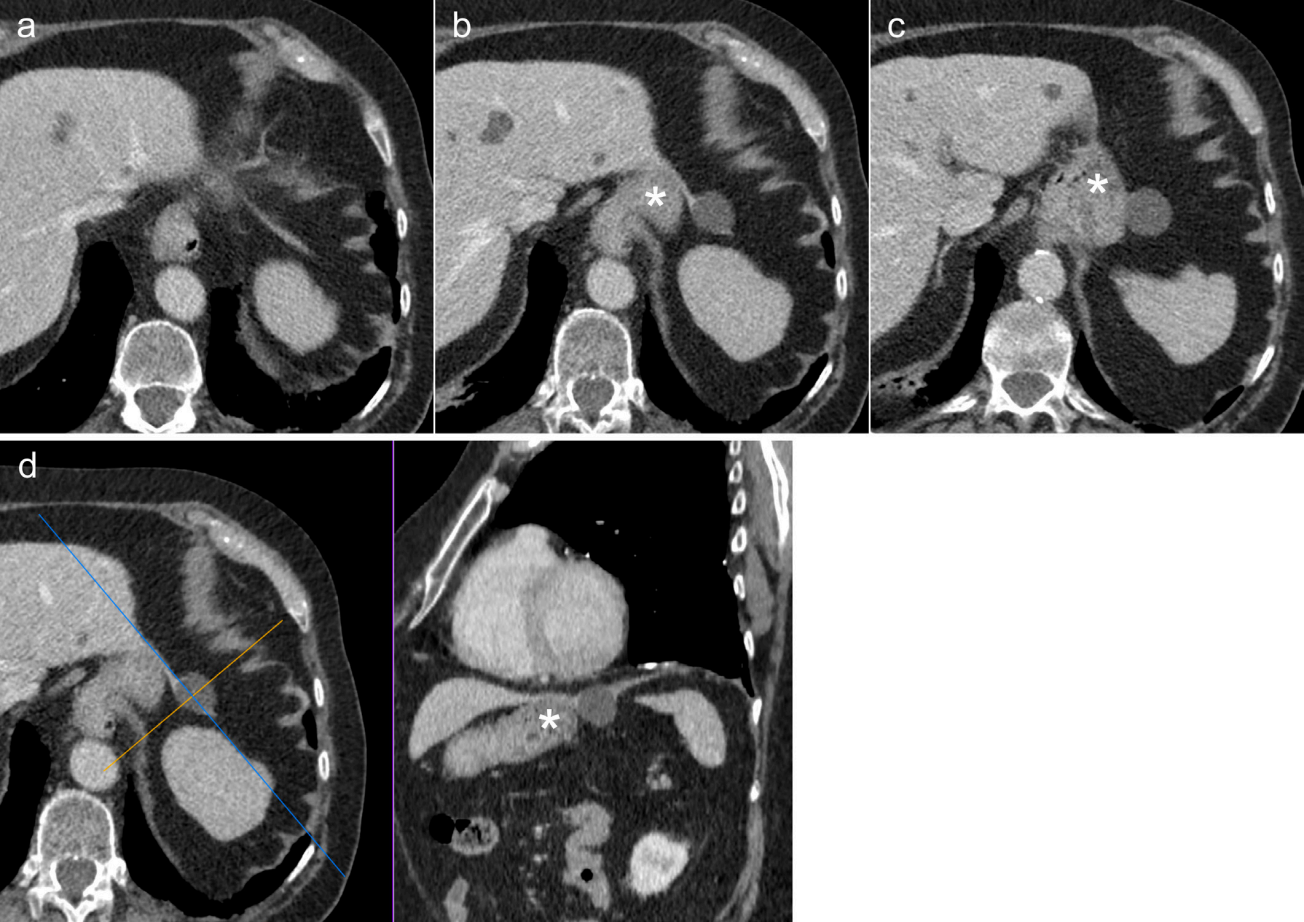
Case 1: 82-year-old male with a simple cyst in the left triangular ligament (LTL). Axial CT slices (cranio-caudal, (**a-c**) of the upper abdomen with contrast in the portal venous phase demonstrate a cystic lesion (arrow) in the left upper quadrant, superolateral to the stomach fundus (asterisk) arising within the LTL (arrowheads). Reconstructed oblique images along the plane of the left triangular ligament (**d**) demonstrate the LTL extending from the left lobe of the liver to the left hemidiaphragm. The cyst is seen arising from the ligament, as shown by the obtuse angle the ligament makes with the cyst.

The falciform ligament is continuous superiorly with the coronary ligament, which is continuous with the left triangular ligament (LTL) at the lateral aspect of the left lobe of the liver (Figure 1). The LTL is a peritoneal fold that connects the liver to the left hemi-diaphragm, crossing the left upper quadrant of the abdomen passing close to the gastric fundus; this is best illustrated in Figures 1 and 2.^1^

At the long free edge of this ligament is the appendix fibrosa hepatis (AFH), which is atrophied liver tissue, which commonly contains bile ducts and remnants of hepatic tissue. In a sample of Japanese cadaveric livers, 23/27 had complete portal tracts with hepatocytes in the proximal third of the AFH.^1^ In a sample of Greek cadaveric livers, 13/80 (16%) had bile ducts in the LTL.^2^ Multiple histological studies also describe ‘irregular islands of hepatic tissue’ specifically within the LTL.^3–6^

Given the embryological origins of the LTL and AFH, we hypothesised that lesions within both structures were likely to be hepatic in origin. Lesions within these structures are rare and to date there has not been a review of the literature of lesions found in the LTL or AFH. We present two cases of lesions within the LTL and have undertaken a systematic review of all the available case reports on the LTL. The primary objective of the systematic review was to develop a list of pathological processes encountered in the LTL and AFH. This would have implications for both diagnosis and management of lesions in the LUQ.

## Case series

### Case description 1

An 82-year-old male underwent a CT of the chest, abdomen, and pelvis as part of the diagnostic workup for a spinal mass. CT imaging revealed an incidental 26 × 25 mm thin-walled cyst (average 6 Hounsfield Units) in the LUQ of the abdomen (Figure 2). The lesion appeared to originate from the LTL and was thought to be a simple cyst. No further intervention or follow-up was considered necessary.

### Case description 2

**Figure 3.**
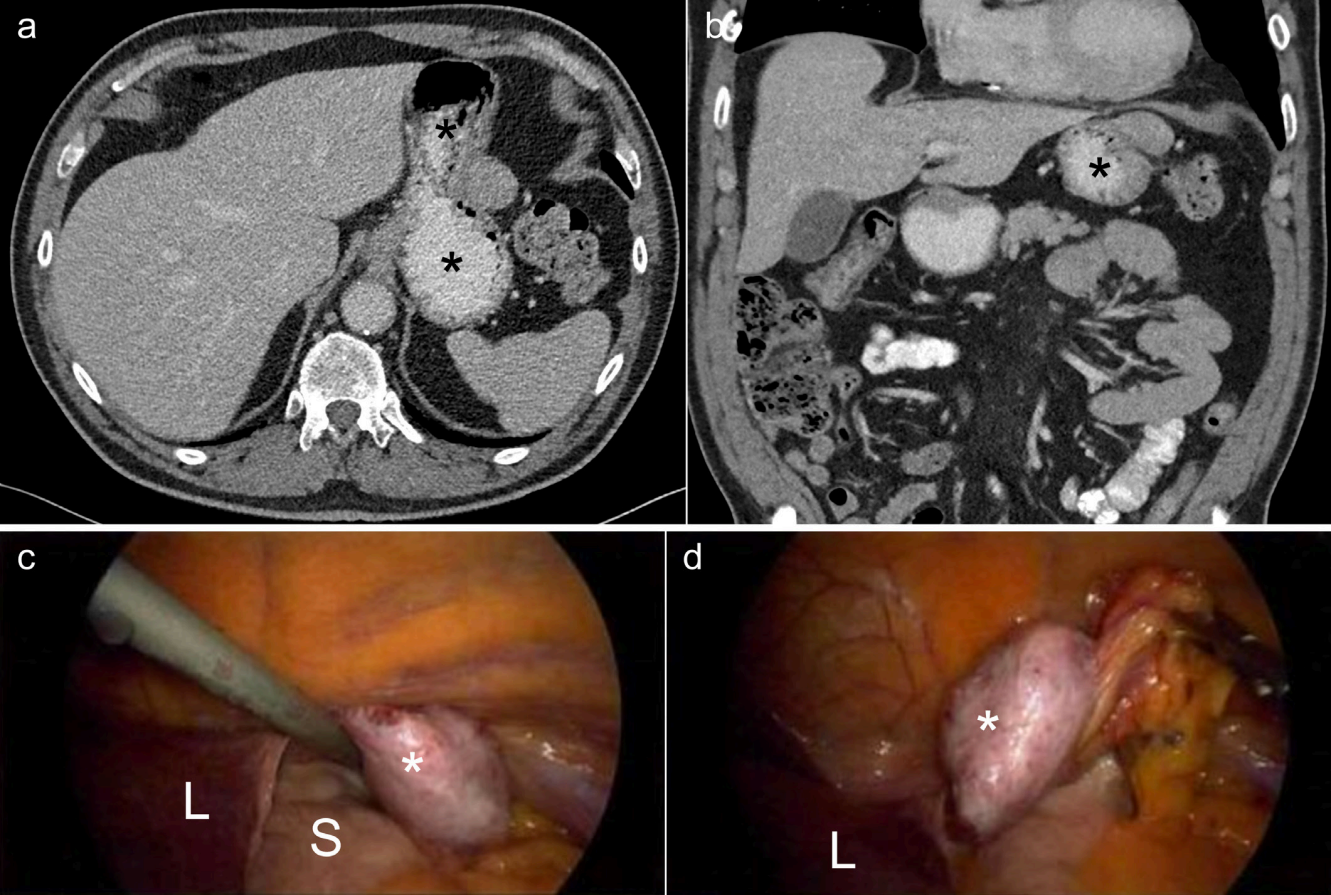
Case 2: a 69-year-old male smoker with a hepatocellular carcinoma in the left triangular ligament. Axial (**a**) and coronal (**b**) CT images with contrast in the portal venous phase demonstrating a soft tissue lesion (arrow) superolateral to the stomach fundus (asterisk). There is no clear involvement of the stomach wall, however this was initially thought to represent a gastrointestinal stromal tumour. Laparoscopic images during surgery demonstrate (**c**) the lesion (asterisk) lifted by attachment to the LTL to demonstrate it is not attached to the stomach (**S**), and (**d**) the lesion attached to the liver (**L**) via the LTL (arrow) being displayed by gentle retraction of attached omental fat.

A 69-year-old male smoker was referred for a CT scan by his primarycare physician on a suspected cancer referral pathway for chronic cough and left lower zone atelectasis on chest X-ray. CT imaging revealed a 27 × 24 mm lesion adjacent to the stomach wall that was initially thought to be a gastrointestinal stromal tumour (GIST), given its location and exophytic appearance (Figure 3). During laparoscopy, the tumour was unexpectedly found to be extraluminal, taking its blood supply from the LTL and some omental fat between the stomach and spleen. Histology revealed that it was a well-differentiated hepatocellular carcinoma (HCC). Re-examination of the CT confirmed the lesion to be attached to the LTL. A biopsy of the background liver demonstrated steatohepatitis with mild periportal and pericellular fibrosis but without bridging fibrosis or cirrhosis. A normal hepatic venous pressure gradient of 2 mm Hg demonstrated that there was no portal hypertension.

## Methods

This systematic review was conducted according to the Preferred Reporting Items for Systematic Reviews and Meta-Analyses (PRISMA).^7^ The review protocol is registered on the international prospective register of systematic reviews (PROSPERO), CRD42022368250.

### Data sources and searches

The search strategy was designed to be comprehensive to capture all the available literature published on the LTL and AFH. The full strategy is listed in Supplementary Material 1.

Supplementary Material 1.

We searched MEDLINE, EMBASE and PubMed databases for studies published until first September 2022. Google Scholar was used to identify manuscripts not in these databases until first September 2022, with the search limited to terms appearing in the title only, as limiting to abstracts was not available. Bibliographies from identified studies were searched for additional relevant studies.

### Study eligibility

All case reports or case series that described a pathological process at the LTL or AFH in humans were included. Publications were not limited by year of publication, language, or study type. Conference abstracts and pre-prints were included where identified.

### Study selection, data extraction and analysis

Duplicates were removed from all abstracts identified from the literature search. Two authors (SA and CC) independently screened all abstracts using the Rayyan platform and excluded ineligible studies against the eligibility criteria.^8^

If author thought either an abstract matched the eligibility criteria or it was unclear whether a study matched eligibility from the abstract alone, that study was included for full-text screening (including any disagreements). From the full texts, SA and CC screened and excluded ineligible studies. All disagreements were resolved with discussion, without the need for arbitration.

Patient demographic data and descriptive details and context of LTL pathology were extracted from the included studies into a summary table by one author (SA) and were reviewed by two other authors independently (RM, CC).

Case reports are inherently anecdotal therefore a risk of bias assessment was not considered appropriate. No further analysis or formal assessment of publication bias was performed.

## Results

### Study selection

**Figure 4.**
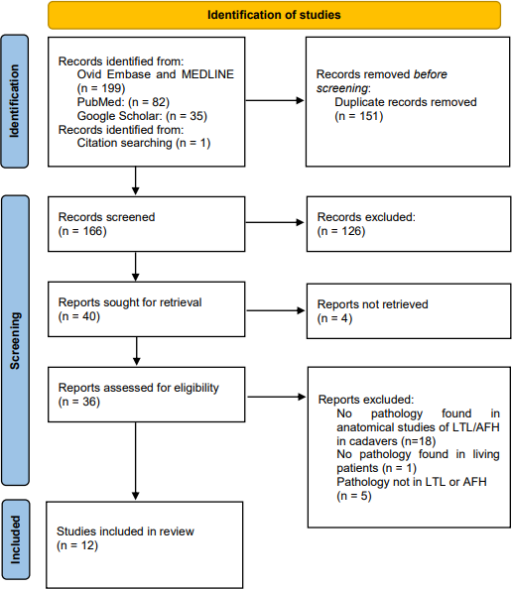
PRISMA flowdiagram.

Database searches and citation searching yielded 166 unique records, of which 40 potentially eligible full texts were assessed; this process is summarised in a PRISMA flow diagram (Figure 4). We were unable to retrieve four records, of which the details can be found in Supplementary Material 2. 26 full texts were excluded for not describing a pathological process in either the LTL or the AFH.

Supplementary Material 2.

PRISMA 2020 Checklist.

### Characteristics of included studies

The above two cases described in this article were included with the 12 cases identified from the systematic review bringing the total cases to 14. There were seven cases of mass lesions in the LTL and seven cases of post-operative bile leaks originating from the LTL. From the available titles and abstracts of the four records not retrieved, one describes a hepatocellular carcinoma in the LTL and three describe post-operative bile leaks (Supplementary Material 2). No case studies described lesions at the AFH.

Every pathology described at the LTL was hepatic in origin (except for a simple cyst, described above in Case 1, for which no histology was deemed necessary). Brief summaries of each case can be found in Table 1.

**Table 1.** Included studies, sorted by pathology category followed by year. LTL = left triangular ligament, HCC = hepatocellular carcinoma, GIST = gastrointestinal stromal tumour

Study	Patient(s)	Pathology	Case summary
Chida, 1992^9^	Male, 59, Japan	HCC at LTL, spontaneously ruptured	Presented with shock, 2.5 litres of blood in the abdominal cavity during emergency laparotomy. No cirrhosis on background liver biopsy.
Kanzaki, 2010^10^	Female, 59, Japan	Moderately differentiated HCC (20 mm) at LTL	CT prompted by a rise in serum AFP from 42 to 2,508 ng ml^−1^ over two years. The HCC was fed by the dorsolateral branch of the hepatic artery and demonstrated hyperenhancement in the arterial phase and washout in the portal venous phase relative to liver parenchyma. Normal liver tissue seen in unaffected parts of the LTL.
Current study, 2022	Male, 69, UK	Well differentiated HCC (27 mm) at LTL	Initially misdiagnosed as a GIST based on CT appearances. Background liver had steatohepatitis but without cirrhosis or portal hypertension.
Current study, 2022	Male, 82, UK	Simple cyst (26 mm) at LTL	Incidental finding, histology not obtained.
Park, 2008^11^	Female, 51, South Korea	Simple hepatic cyst (36 mm)	Misdiagnosed as GIST or gastric duplication cyst on endoscopic ultrasound and CT. Laparoscopic resection revealed that it was a cyst arising from the left lateral segment of the liver.
Poznanski, 1959^12^	Female, 55, USA	Cystic mass lesion (35 mm) of hepatic origin at LTL	Symptoms of epigastric pain, lesion identified on double contrast barium meal and plain radiograph with iatrogenic pneumoperitoneum.
Song, 2015^13^	Male, 36, China	Cavernous haemangioma (20 mm) of hepatic origin at LTL	Misdiagnosed as gastric duplication cyst on CT, with difficulty in locating the tumour intraoperatively. Resection required two attempts at gastroscopy, exploratory laparoscopy, followed by laparotomy.
Rapant, 1950^3^	Male, 51, Czechoslovakia	Post-operative bile leak	LTL transected, bile leak noted and sutured intraoperatively.
Iso, 1996^14^	Male, 61, Japan	Post-operative bile leak	LTL transected unintentionally, with post-operative bile leak and peritonitis. Intraoperative cholangiography was required to identify the aberrant bile duct in the LTL
Takeuchi, 2000^15^	Male, 75, Japan	Post-operative bile leak	LTL transected. Large volume of bile noted in abdominal drain post-operatively. LTL ligated during further exploration.
Kitamura, 2017^16^	Male, 67, Japan	Post-operative bile leak	LTL mobilised. Bile leak noticed and ligated intraoperatively.
Yildiz, 2018 ^17^	Female, 67, Turkey	Post-operative bile leak	LTL transected. 150 ml bile leak over 24 h. One 3 mm diameter aberrant bile duct sutured, with no further leak.
Fukui, 2021^18^	Male, 91, Japan	Post-operative bile leak	Bile leak shortly after endoscopic sphincterotomy, with biliary peritonitis. Intraoperative cholangiography identified a ruptured bile duct in the LTL.
Shah, 2022^19^	Female, 57, USA	Post-operative bile leak	LTL mobilised using electrocautery. Bile leak noted post-operatively, with small bile duct sutured.

### Mass lesions

Included studies were published between the years 1959 and 2022, the age at presentation ranged from 36 to 82, and 3/7 (43%) were female. In 3/7 (43%) of cases, mass lesions were initially misdiagnosed on CT imaging as tumours of gastric origin.

### Post-operative bile leaks

Included studies were published between the years 1950 and 2022, the age at presentation ranged from 51 to 91, and 2/7 (29%) were female. In all cases, bile leaks were observed from aberrant bile ducts in the LTL. In the majority of cases, this was from a cut surface of the LTL (6/7, 86%), and in one case from a ruptured bile duct shortly following sphincterotomy.^18^ All leaks were resolved by ligating the LTL.

## Discussion

All cases except one described LTL pathology of hepatic origin. Half of them described mass lesions while the other half described postoperative bile leaks. Given our broad search strategy, this likely represents the majority of all the reported literature available on pathology in the LTL or the AFH, suggesting that pathology at this site is rare.

Three (43%) mass lesions were initially misdiagnosed as tumours of gastric origin - the anatomical course of the LTL from the left lateral segment of the liver towards the left hemi-diaphragm, means that lesions here can indent the stomach. The burden of misdiagnosis caused diagnostic delay and additional unnecessary procedures in two out of three of these cases. One of these cases resulted in additional two endoscopies with endoscopic ultrasound, laparoscopy and ultimately a laparotomy to determine that the lesion originated from the LTL.^13^

### Implications for diagnostic imaging

Radiologists should consider whether lesions could be arising from the LTL when reviewing masses seen in the LUQ of the abdomen. It is easier to determine if LUQ mass lesions arise from the LTL when the LTL is visualised separate to the stomach (Case 1, Figure 2). In practice however, the LTL is often hard to visualise and differentiate from the stomach because it is a thin fold of peritoneum that is variable in length and position. It is also continuous with the left hemidiaphragm and therefore prone to respiratory motion artefact.

All three cases of diagnostic confusion arose from lesions which were initially considered to be gastric in origin due to their close relation to the stomach. It may not be possible to differentiate a gastric lesion from a LTL lesion on imaging features alone; however, an LTL lesion should be considered in the differential diagnosis of a seemingly gastric lesion if any of the following features are present:

- The lesion appears predominantly ‘exophytic’ or external to, but in close contact with the stomach fundus.
- A linear structure (*i.e.,* the LTL) is seen to run from the lesion to either the diaphragm or the left lobe of the liver as demonstrated in Case 2 (Figure 3).
- The LTL is not visualised separately to the lesion.
- It is possible to delineate the stomach wall separate to the lesion (suggesting no involvement of the stomach wall).

Although one HCC case in this review demonstrated the characteristic enhancement pattern of early enhancement and washout,^20^ characterising LTL lesions may be challenging as we cannot assume the blood flow dynamics are the same as if the lesion were seen within the liver parenchyma. Of note, none of the patients with HCC in this review had background cirrhosis.

### Strengths and limitations

To our knowledge, this is the first systematic review of pathology encountered at the LTL and AFH. A limitation of the design of this study is that the overall level of evidence is low as case reports are inherently anecdotal, precluding any risk of bias assessment. Only 14 cases were identified, despite having a broad search strategy. No formal assessment of publication bias was possible. No case report described AFH pathology, although clinically the AFH is rarely distinguished from the LTL so some of the LTL lesions described may have originated from the AFH instead.^1^

Four case reports were not able to be retrieved. The titles and abstracts describe one HCC and three postoperative bile leaks which suggest that the conclusions would be unlikely to change had they been included.

## Conclusion

Our initial hypothesis was that LTL and AFH lesions are likely hepatic given that cadaveric studies have demonstrated remnant hepatic tissue in both structures. All lesions observed were considered to have originated from the LTL, which appears to support this hypothesis. Whilst the quality of evidence from each individual case report is low, to reduce the burden of diagnostic delay and unnecessary procedures, we would suggest that these observations support the addition of hepatic lesions to the differential diagnosis of LUQ lesions, particularly if it is difficult to exclude attachment to the LTL.
