# Supplementary material for: Left triangular ligament lesions are likely hepatic in origin
Source: Br J Radiol. 2023 Oct 3;96(1152):20230231. doi: 10.1259/bjr.20230231 (PMC10646653; doi:10.1259/bjr.20230231)
Supplement: Supplementary Material 1. [file bjr.20230231.suppl-01.docx]

# Electronic Supplementary Material

## Supplemental material 1: Search Strategy

**Ovid MEDLINE, EMBASE search strategy, 01/09/2022: 199 results**

1. left ajd2 triangular adj2 ligament.mp.
2. ligamentum triangulare.mp.
3. appendix fibrosa hepatis.mp.
4. fibrous appendix.mp.
5. coronary ligament.mp.
6. 1 or 2 or 3 or 4 or 5

**PubMed search strategy, 01/09/2022: 82 results**

("left triangular ligament") OR ("left triangular hepatic ligament") OR ("left hepatic triangular ligament") OR ("ligamentum triangulare") OR ("appendix fibrosa hepatis") OR ("fibrous appendix") OR ("coronary ligament")

**Google Scholar search strategy, 01/09/2022: 35 results**

allintitle: "left triangular ligament" OR "left triangular hepatic ligament" OR "left hepatic triangular ligament" OR "ligamentum triangulare" OR "appendix fibrosa hepatis" OR "fibrous appendix" OR "coronary ligament"
