# Supplementary material for: Left triangular ligament lesions are likely hepatic in origin
Source: Br J Radiol. 2023 Oct 3;96(1152):20230231. doi: 10.1259/bjr.20230231 (PMC10646653; doi:10.1259/bjr.20230231)
Supplement: Supplementary Material 2. [file bjr.20230231.suppl-02.docx]

## Supplemental material 2: Records not retrieved

| Authors | Title | Year | Journal |
| --- | --- | --- | --- |
| J., Waldschmidt; R., Haring; S., John; E., Kourias; R., De Pena Perez; | Spontaneous perforation of dystopic bile ducts in the triangular ligaments of the liver | 1971 | Zentralblatt fur Chirurgie - Volume 96, Issue 27, pp. 902-907 |
| M., Balija; M., Huis; J., Bubnjar; | Accessory biliary duct and postoperative biliary leak | 1998 | Croatian Journal of Gastroenterology and Hepatology - Volume 7, Issue 3, pp. 71-75 |
| Hiroaki, S; Shigeki, W; journal, Y Katsuhiko | Aberrant Bile Duct in the Left Triangular Ligament of the Liver (Case Report) | 2017 | Jikeikai medical journal |
| Y., Kurata; T., Hinoda; S., Arizono; H., Ueda; | A case report of ectopic hepatocellular carcinoma arising in the left triangular ligament of the liver | 2013 | Japanese Journal of Clinical Radiology |
